# Trends in South Korean Medical Device Development for Attention-Deficit/Hyperactivity Disorder and Autism Spectrum Disorder: Narrative Review

**DOI:** 10.2196/60399

**Published:** 2024-10-15

**Authors:** Yunah Cho, Sharon L Talboys

**Affiliations:** 1 Division of Public Health, Department of Family and Preventive Medicine University of Utah Asia Campus Incheon Republic of Korea; 2 Division of Public Health, Department of Family and Preventive Medicine University of Utah School of Medicine Salt Lake City, UT United States

**Keywords:** ADHD, attention-deficit/hyperactivity disorder, ASD, autism spectrum disorder, medical device, digital therapeutics

## Abstract

**Background:**

Attention-deficit/hyperactivity disorder (ADHD) and autism spectrum disorder (ASD) are among the most prevalent mental disorders among school-aged youth in South Korea and may play a role in the increasing pressures on teachers and school-based special education programming. A lack of support for special education; tensions between teachers, students, and parents; and limited backup for teacher absences are common complaints among Korean educators. New innovations in technology to screen and treat ADHD and ASD may offer relief to students, parents, and teachers through earlier and efficient diagnosis; access to treatment options; and ultimately, better-managed care and expectations.

**Objective:**

This narrative literature review provides an account of medical device use and development in South Korea for the diagnosis and management of ADHD and ASD and highlights research gaps.

**Methods:**

A narrative review was conducted across 4 databases (PubMed, Korean National Assembly Library, Scopus, and PsycINFO). Journal articles, dissertations, and government research and development reports were included if they discussed medical devices for ADHD and ASD. Only Korean or English papers were included. Resources were excluded if they did not correspond to the research objective or did not discuss at least 1 topic about medical devices for ADHD and ASD. Journal articles were excluded if they were not peer reviewed. Resources were limited to publications between 2013 and July 22, 2024.

**Results:**

A total of 1794 records about trends in Korean medical device development were categorized into 2 major groups: *digital therapeutics* and *traditional therapy*. Digital therapeutics resulted in 5 subgroups: *virtual reality and artificial intelligence*, *machine learning and robot*, *gaming and visual contents*, *eye-feedback and movement intervention*, and *electroencephalography and neurofeedback*. Traditional therapy resulted in 3 subgroups: *cognitive behavioral therapy and working memory*; *diagnosis and rating scale*; and *musical, literary therapy, and mindfulness-based stress reduction*. Digital therapeutics using artificial intelligence, machine learning, and electroencephalography technologies account for the biggest portions of development in South Korea, rather than traditional therapies. Most resources, 94.15% (1689/1794), were from the Korean National Assembly Library.

**Conclusions:**

Limitations include small sizes of populations to conclude findings in many articles, a lower number of articles discussing medical devices for ASD, and a majority of articles being dissertations. Emerging digital medical devices and those integrated with traditional therapies are important solutions to reducing the prevalence rates of ADHD and ASD in South Korea by promoting early diagnosis and intervention. Furthermore, their application will relieve pressures on teachers and school-based special education programming by providing direct supporting resources to students with ADHD or ASD. Future development of medical devices for ADHD and ASD is predicted to heavily rely on digital technologies, such as those that sense people’s behaviors, eye movement, and brainwaves.

## Introduction

### Background

Attention-deficit/hyperactivity disorder (ADHD) and autism spectrum disorder (ASD) are some of the most prevalent mental disorders among school-aged youth in South Korea. Insufficient support for those with ADHD or ASD affects their delayed improvement, and this circumstance may play a role in the increasing pressures on teachers and school-based special education programming. As teachers are the second most important people who impact children’s early diagnosis and intervention [1], teachers are under increasing pressure in South Korea from parents and substandard special education resources, leading them to protest [2]. The protests were prompted by the news of a teacher who resorted to suicide over excessive complaints from demanding parents while also trying to manage students [3]. Sadly, this tragedy was followed by several more incidents of teacher suicides [4]. The lack of support for special education; tensions between teachers, students, and parents; and the lack of backup for teacher absences are common complaints among Korean educators [4]. New innovations in technology to screen and treat ADHD and ASD may offer some relief to students, parents, and teachers through earlier and efficient diagnosis; access to treatment options; and ultimately, better-managed care and expectations.

### Prevalence of ADHD and ASD

ADHD is recognized by an ongoing pattern of inattention and hyperactivity-impulsivity that interferes with development or functioning [5]. ASD is defined as a developmental and neurological disorder that affects how people communicate with others, interact, behave, and learn [6]. The number of patients with ADHD in South Korea has consistently increased, and the total number has risen by 250% in 2022 [7]. Among this entire population, people aged between 0 and 19 years accounted for the majority of cases, ranging from 57% to 85% from 2018 to 2022 [8]. The prevalence of ASD in 2021 was 12.8%, which translated to roughly 32,000 individuals [9]. The rate has increased by 4.3% since 2010 [9]. According to the database of registered people with developmental disabilities in June 2021, the Ministry of Health and Welfare of South Korea announced that 56.7% of the population with ASD were young individuals aged between 0 and 19 years [10].

### Objective

To set up improved special education systems for young people with ADHD or ASD, experts claim that innovational medical devices for ADHD and ASD are crucial to treating them in a timely and proper manner [11,12]. While diverse types of medical devices exist, including devices for assessment, screening, and training, few studies have examined the use of these medical devices in South Korea or trends in the development of new devices for ADHD and ASD in South Korea. This study provides a review of the literature focused on gaps in the research related to medical device use and development in South Korea for the diagnosis and management of ADHD and ASD.

## Methods

### Search Strategy

A narrative review was conducted to examine the trends in Korean medical device development focusing on medical equipment for ADHD and ASD, using the National Assembly Library, PubMed, Scopus, and PsycINFO. The detailed search terms were presented in the *Search Strategies* section in Multimedia Appendix 1. Data and studies were retrieved and reviewed after screening years and language. Key search terms included: *ADHD*, *ASD* or *autism*, *early*, *diagnosis*, *treatment*, *screening*, *medical device*, *intervention*, and *training*. The list of references from the 4 databases was cross-checked to identify duplicates.

### Eligibility Criteria

Journal articles and dissertations were included if they discussed diverse types of medical devices for ADHD and ASD, were peer reviewed, and were published in 2013 or later. Government research and development project reports were also included if they discussed relevant topics and were published in 2013 or later. Only Korean or English papers were included. The expected outcome from the included sources was updated information on Korean medical equipment for ADHD and ASD and an emphasis on examining the trends in Korean medical equipment for ADHD and ASD. Non–peer-reviewed interview articles were also included.

Resources were excluded if they did not correspond to the research objective or did not discuss at least 1 topic about medical devices for ADHD and ASD in the title or abstract. Journal articles were excluded if they were not peer reviewed, published before 2013, or written other than Korean or English. Detailed eligibility criteria for study inclusion are described in Multimedia Appendix 1.

## Results

### Selection of Sources of Evidence

**Figure 1:**
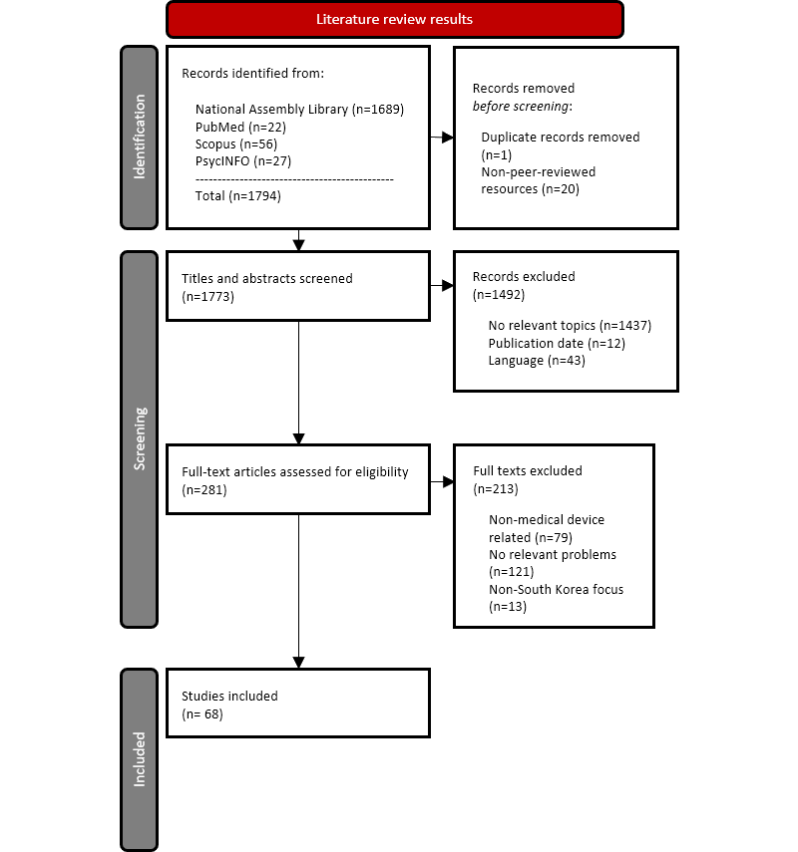
Flow diagram of narrative review describing the selection of sources of evidence.

The search identified 94.15% (1689/1794) records through the National Assembly Library, 1.23% (22/1794) records through PubMed, 3.12% (56/1794) records through Scopus, and 1.5% (27/1794) records through PsycINFO (Figure 1). Of the total 1794 records, 1 (0.1%) duplicate record was found, and 20 (1.1%) records were removed after non–peer-reviewed resources were screened. Of the remaining 1773 full-text records, 84.1% (n=1492) were excluded based on their content. Among the remaining 281 records, 213 were excluded: 37.1% (79/213) of resources were unrelated to medical devices, 56.8% (121/213) were irrelevant problems, and 6.1% (13/213) were non–South Korea focused. Thus, 24.1% (68/281) of records were included in this paper. Figure 1 depicts a flow diagram describing the selection of sources of evidence.

### Synthesis of Results

#### Overview

**Figure 2:**
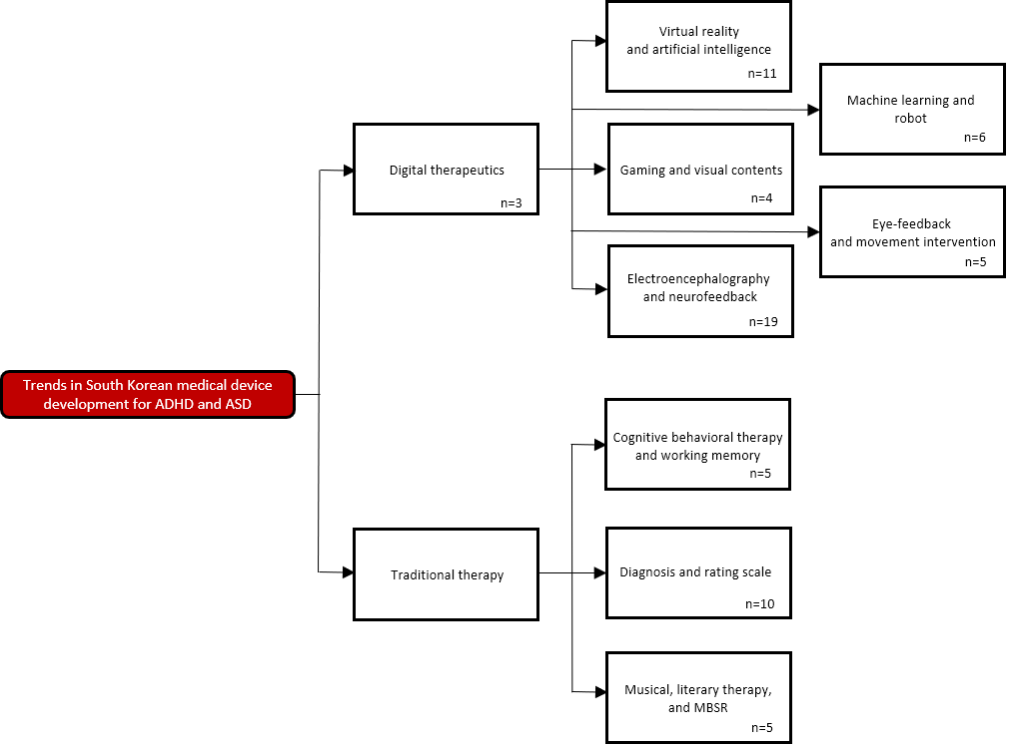
Nine groups of the trends in Korean medical device development for attention-deficit/hyperactivity disorder (ADHD) and autism spectrum disorder (ASD). MBSR: mindfulness-based stress reduction.

After a review of the records, 9 categories were developed post hoc to describe trends in Korean medical device development (Figure 2). The 9 groups included *digital therapeutics*; *virtual reality (VR) and artificial intelligence (AI)*; *machine learning and robot*; *gaming and visual contents*; *eye-feedback and movement intervention*; *electroencephalography and neurofeedback*; *cognitive behavioral therapy (CBT) and working memory*; *diagnosis and rating scale*; and *musical, literary therapy, and mindfulness-based stress reduction (MBSR)*. These 9 groups were recategorized into 2 big groups: *digital therapeutics* and *traditional therapy*.

Table 1 summarizes the selected resources on the trends in Korean medical device development for ADHD and ASD.

**Table 1:** Overview of the selected resources.

Study, year	Title	Country	Type
Choi [13], 2022	Study of textbooks on the principles of digital therapeutics to respond to ADHD and digital drama	South Korea	Doctoral dissertation
Lee [14], 2022	The efficacy of digital therapeutics for the treatment of attention deficit hyperactivity: a systematic review and meta-analysis	South Korea	Master’s thesis
Son et al [15], 2023	Current status and outlook for digital therapeutics	South Korea	Research report
Rashid et al [16], 2024	Power of alignment: exploring the effect of face alignment on ASD diagnosis using facial images	Malaysia	Peer-reviewed article
Kim [17], 2019	To strive for the universalization of virtual reality therapy programs	South Korea	Interview
Korea Electronics Technology Institute [18], 2020	VR/AR platform technology based on bio-signal for mental health of kids/silver generation	South Korea	Government R and D^a^ project report
Megerian et al [19], 2022	Evaluation of an artificial intelligence-based medical device for diagnosis for autism spectrum disorder	United States	Peer-reviewed article
Son [20], 2022	Towards standardizing attention-deficit/hyperactivity disorder diagnosis- a virtual reality, artificial intelligence application	South Korea	Master’s thesis
Park et al [21], 2019	Design and implementation of VR-based life care contents for attention deficit hyperactivity disorder (ADHD)	South Korea	Peer-reviewed article
Ryu [22], 2022	Implications of VR-based psychotherapeutic effects for ADHD and CD among adolescents	South Korea	Peer-reviewed article
Ryu and Hwang [23], 2021	Artificial intelligence analysis of biosignals for automated detection and automated diagnosis of ADHD and CD	South Korea	Peer-reviewed article
Voss et al [24], 2019	Effect of wearable digital intervention for improving socialization in children with autism spectrum disorder a randomized clinical trial	United States	Peer-reviewed article
Yoo [25], 2020	virtual reality based digital therapeutics system for diagnosing attention-deficit hyperactivity disorder	South Korea	Master’s thesis
Yonsei University Office of Research Affairs [26], 2019	Development of mobile VR neurocognitive battery and establishment of database, implementation of AI-based early diagnosis/prevention system for cognitive control vulnerable groups utilizing digital representation modeling	South Korea	Government R and D project report
Imbiriba et al [27], 2023	Wearable biosensing to predict imminent aggressive behavior in psychiatric inpatient youths with autism	United States	Peer-reviewed article
Kim et al [28], 2016	Exploring the applicability of Tele-presence robot intervention for at-risk children with ADHD	South Korea	Peer-reviewed article
Lee [29], 2022	Development of a contract-less sensing system and a classifier using deep learning for robot-based ADHD screening	South Korea	Doctoral dissertation
Lee et al [30], 2021	Development of a machine-learning predictive model for first-grade children at risk for ADHD	South Korea	Peer-reviewed article
Shin et al [31], 2018	Exploring the performance difference on the active based task with a robot for ADHD screening	South Korea	Peer-reviewed article
Yeom [32], 2018	Supervised classification of childhood ADHD using robot-assisted tests	South Korea	Master’s thesis
Jung [33], 2022	ADHD can be treated like playing a game in daily life	South Korea	Interview
Lee and Lim [34], 2018	A study on the effect of communication functional board game on self-control, self-esteem, family function and peer relationship of ADHD children	South Korea	Peer-reviewed article
Park [35], 2019	To improve the concentration of ADHD children study on functional games	South Korea	Master’s thesis
Sungkyunkwan University Cooperation Center [36], 2020	A study on the therapeutic applications of digital games	South Korea	Government R and D project report
Kim [37], 2018	Development of movement intervention visualization contents to improve behavior of ASD and ADHD	South Korea	Master’s thesis
Kim [38], 2019	The characteristic of attentional networks in sluggish cognitive tempo: the effect of eye-feedback training on orienting attention in individuals with SCT	South Korea	Doctoral dissertation
Sandbank and Cascio [39], 2018	Using a motion-tracking device to facilitate motion control in children with ASD for neuroimaging	United States	Peer-reviewed article
Yoo and Kim [40], 2015	A preliminary study on the development of the focus reaction time tests	South Korea	Peer-reviewed article
Yoo et al [41], 2024	Development of an innovative approach using portable eye tracking to assist ADHD screening: a machine learning study	South Korea	Peer-reviewed article
Alhassan et al [42], 2023	Energy-efficient EEG-based scheme for autism spectrum disorder detection using wearable sensors	United States	Peer-reviewed article
Bhattacharyya et al [43], 2022	Integration of electroencephalogram (EEG) and motion tracking sensors for objective measure of attention-deficit hyperactivity disorder (MAHD) in preschoolers	United States	Peer-reviewed article
Hong et al [44], 2013	Development of brain imaging diagnosis and brain-based training programs for ADHD students	South Korea	Government R and D project report
Hong et al [45], 2014	Validation of the effectiveness of brain-based training programs for ADHD students	South Korea	Government R and D project report
Kang [46], 2013	Brain music as a potential tool for diagnosing attention-deficit/hyperactivity disorder (ADHD)	South Korea	Master’s thesis
Kim [47], 2017	The effects of neurofeedback training and executive function improvement programs on attention and brain function quotient of elementary school children	South Korea	Master’s thesis
Kim [48], 2021	Machine learning-based EEG classification for assisting the diagnosis of ADHD in children	South Korea	Peer-reviewed article
Kim [49], 2022	Deep learning approach on the improvement of diagnosing ADHD with fMRI	South Korea	Master’s thesis
Kim et al [50], 2015	Clinical significance for neurofeedback training of children with attention-deficit/hyperactivity disorder	South Korea	Peer-reviewed article
Kim et al [51], 2022	The classification scheme of ADHD for children based on the CNN model	South Korea	Peer-reviewed article
Lee [52], 2013	The effects of the neurofeedback training on the attention in adolescents with autism spectrum disorder	South Korea	Master’s thesis
Lee [53], 2020	Effects of neurofeedback brain wave training on the attention concentration and language development of children delayed in language development	South Korea	Master’s thesis
Lee [54], 2022	The effect of EEG training through neurofeedback on attention and pragmatic language ability in children with ADHD prone language delay	South Korea	Master’s thesis
Nam [55], 2016	Effect of neurofeedback based robotic invention education of attention ability of ADHD children	South Korea	Peer-reviewed article
Nam and Mun [56], 2015	Development of neurofeedback based robotic invention education program for ADHD children	South Korea	Peer-reviewed article
Ryu [57], 2015	Effects of neurofeedback training on EEG, continuous performance task, and ADHD symptoms in ADHD in ADHD-prone college students	South Korea	Master’s thesis
Ryu [58], 2021	A study on the clinical usefulness of EEG and QEEG measurements for the diagnostic criteria of ADHD	South Korea	Peer-reviewed article
Siddharth et al [59], 2019	A wearable multi-model biosensing system toward real-world applications	United States	Peer-reviewed article
Yun and Kwack [60], 2015	The treatment effect of neurofeedback training on executive function in attention-deficit hyperactivity disorder	South Korea	Peer-reviewed article
An et al [61], 2016	Cognitive behavioral therapy for college students with ADHD tendencies	South Korea	Peer-reviewed article
Hong et al [62], 2015	Development of working memory training program for ADHD children and effectiveness verification	South Korea	Peer-reviewed article
Chang and Park [63], 2020	Development and application of the working memory improvement program for children with ADHD in the first grade elementary school	South Korea	Peer-reviewed article
Lee [64], 2019	The effects of self-monitoring cognitive functions training program on the attention-concentration ability and the hyperactivity of the children with ADHD tendency	South Korea	Peer-reviewed article
Park et al [65], 2015	Effects of cognitive behavioral therapy on attention deficit hyperactivity disorder among school-aged children in Korea	South Korea	Peer-reviewed article
Kang et al [66], 2015	Development of Korean adult ADHD rating scale	South Korea	Peer-reviewed article
Kim [67], 2016	(The) clinical utility of K-CBCL 6-18 in diagnosing ADHD: focused on children with psychological disorder in Child Welfare Institution	South Korea	Master’s thesis
Lee [68], 2015	Current status and future improvement of the Korea ADHD rating scale-IV (K-ARS-IV)	South Korea	Peer-reviewed article
Lee [69], 2017	A review on the diagnosis of ADHD for special education	South Korea	Peer-reviewed article
Lee [70], 2020	A review of diagnosis and evaluation procedure for the child and adolescent with attention deficit hyperactivity disorder	South Korea	Peer-reviewed article
Lee et al [71], 2015	Clinical utility of the Korean version of CBCL6-18 in the diagnosis of attention-deficit hyperactivity disorder	South Korea	Peer-reviewed article
Lee et al [72], 2016	The guideline of diagnosis and treatment of attention-deficit hyperactivity disorder: developed by ADHD Translational Research Center	South Korea	Peer-reviewed article
Lee et al [1], 2014	A study on agreement between parent’s and teacher’s ratings according to ADHD screening	South Korea	Peer-reviewed article
National Research Foundation of Korea [73], 2016	Success in quantifying the level of attention and concentration through meditation and exercise [electronic data]: expected to be used in diagnostic tests for ADHD, depression, and dementia in children	South Korea	News release
Park [74], 2015	Clinical application of advanced test of attention as a diagnostic tool in children with attention-deficit/hyperactivity disorder	South Korea	Doctoral dissertation
Cho [75], 2023	Development of rhythm-based music intervention protocols through timing control in children with ADHD	South Korea	Doctoral dissertation
Choi [76], 2019	Development of a music program for improvement of the mental concentration and human relationship using Carl Orff’s pedagogics: centered about the ADHD students	South Korea	Master’s thesis
Kim [77], 2016	The effects of literary therapy program based on SST by using picture cards on ADHD of adolescents for EBD	South Korea	Peer-reviewed article
Kim [78], 2016	Effects of mindfulness-based stress reduction (MBSR) program on attention, perceived stress, and anxiety on attention-deficit/hyperactivity disorder (ADHD) prone university students	South Korea	Master’s thesis
Son [79], 2022	A study on development of diagnostic assessment tools of music therapy in children with attention deficit hyperactivity disorder	South Korea	Doctoral dissertation

^a^R and D: research and development.

#### Digital Therapeutics

Digital therapeutics is a broad category that refers to high-quality software, that is, digital technologies, including AI, VR, augmented reality (AR), apps, and wearable devices, that provide evidence-based therapeutic interventions to patients to prevent, manage, or treat medical disorders or diseases [15]. Digital therapeutics is one of the promising methods of intervention, treatments, and diagnosis for ADHD and ASD in South Korea. Two dissertations [13,14] and 1 research report [15] described digital therapeutics. Several specific types of digital therapeutics, such as AI, machine learning, and VR, will be further discussed in detail in other groups later.

The digital health care market is consistently growing in South Korea, especially since the COVID-19 pandemic has facilitated web-based treatments and health communication. The South Korean government has suggested diverse policies to promote the digital health care industry. In April of 2022, 10 digital therapies were approved by the Ministry of Food and Drug Safety of the Republic of Korea to conduct clinical trials [15]. However, none of them have been approved as a medical device [15]. Although the Korean digital therapeutics industry is in a beginning stage of development, the digital therapeutics market is highly promising in South Korea given the policy support and attention from the South Korean government.

For instance, digital therapeutics is applied to treatments and education for students with ADHD [13,14]. Digital therapeutics is emphasized as a new treatment approach for children and adolescents with ADHD. A substantial improvement was found in groups using digital therapeutics compared with control groups [14]. Educational materials about digital therapeutics were also highlighted for elementary school teachers educating students with ADHD. The use of Korean medical devices for ADHD and ASD reflects the attention to educating teachers about the importance and functions of digital therapeutics, as well as its direct application to those with ADHD or ASD.

#### VR and AI

VR and AI are categorized into digital therapeutics. Both technologies are promising tools that recent studies have highlighted their potential [19,20]. VR and AI were mentioned in 11 resources. Two government research and development project reports [18,26], 6 peer-reviewed articles [10-15,18-23,26], 2 dissertations [16,20,24,25], and 1 interview [17] described medical devices for ADHD and ASD using VR and AI.

Although the fundamental treatment method for ADHD is medication, behavioral problems are treated by CBT [17]. However, traditional CBT has limited accessibility in clinical settings due to additional time to visit hospitals, health care personnel, and relevant resources [17]. In this circumstance, VR technology has a big advantage in solving these limitations by enabling real-time simulations and virtual training [17]. VR-based diagnosis of ADHD is also considered to have accurate and objective results given that the model is based on VR settings, while the traditional diagnosis relies on verbal interviews [25]. Furthermore, virtual social interactions allow repetitive practice for anger recognition, anger regulation, and social problem solving [22]. This VR-based training helps control their aggressive and impulsive behaviors [22]. Some recent studies also indicated that VR-based interventions for ADHD can prevent potential crime, especially for young people with ADHD [22,23]. An AI-automated diagnosis system for diagnostic classification and automated detection based on the biosignals of ADHD was introduced for the prediction, suppression, and prevention of adolescent recidivism [23].

To enhance the efficiency of treatment and diagnosis of ADHD and ASD, VR technologies are often integrated with AI to predict, analyze, and define different types of data from patients and users [19,20]. For example, a device using a gradient-boosted decision tree algorithm was evaluated to test the accuracy of its AI-based software when health care providers diagnose ASD in children aged between 18 and 72 months [19]. The study found that an increased number of children with ASD were able to be diagnosed in a primary care setting, potentially promoting early intervention and treatment [19]. In addition, the combination of a deep learning algorithm and facial images is a novel approach in the diagnosis of ASD [16]. Given that ASD is marked by impaired neurological development, the human face provides insights into brain structure and function [16]. Consequently, facial features can serve as an important biomarker for diagnosing ASD [16]. This idea is also applied to a wearable digital intervention that promoted emotion recognition and facial engagement [24]. Researchers found that children with ASD who wore superpower glasses showed significant improvements on socialization by providing social cues and detecting facial expressions [24].

Following the trends of the combined technologies with VR and AI, research and government research and development projects have studied possible medical device models for people with ADHD or ASD [18,21,25,26]. The Korea Electronics Technology Institute conducted a project to develop VR and AR platform technology based on biosignals for the mental health of kids and silver generation [18]. In total, 7 types of VR, 6 types of AR mental health content, a mental-care cloud platform, wireless transfer technology for 4K video streaming, and systems to measure and analyze biosignals were developed throughout the project [18]. These VR and AR technologies can be broadly applied to mental health VR and AR platform services at mental-health clinics in South Korea [18]. This application also positively impacts the Korean web content industry beyond the Korean medical device industry.

Medical VR and AI technologies were interweaved with IT and life care content markets [21]. Regarding ADHD treatments, contents and systems using immersive and vivid exposure in virtual settings have been actively tried [21]. The system virtually provides the actual circumstances where people with ADHD can be trained through sensory, cognitive, and linguistic simulations [21]. With a similar purpose, the Office of Research Affairs at Yonsei University conducted a project to develop mobile VR neuropsychological batteries and an AI-based database of early diagnosis and promotion systems using digital phenotypic modeling [26]. The developed device was based on a multilayer platform integrating emotions, social ability, and neurological information [26]. Both devices target vulnerable populations with limited access to traditional treatments for ADHD.

The overall trends of Korean medical devices for ADHD and ASD concentrate on improving the current conditions of the medical device application and its use [25,26]. While most of the traditional approaches require personnel, physical resources, and travel time, VR and AI–based medical devices minimize the requirements.

#### Machine Learning and Robot

Machine learning and robot-based medical devices for ADHD and ASD are also included in the category of digital therapeutics. They were found in 6 resources. Two dissertations [29,32] and 4 journal articles [27,28,30,31] addressed its trends.

The importance of early diagnosis is highlighted in many studies about ADHD and ASD [30,41]. To facilitate early diagnosis and ADHD screening, machine learning and robot-based technologies are used as a promising tool. A machine learning predictive model is one of the solutions to increase the accuracy of ADHD prediction [30]. As a longitudinal predictive model, several types of machine learning analysis were applied to predict the future and classify findings, such as supervised learning, random forest, gradient boosting, and neural network models [30]. This model identified that children who showed specific risk indicators during infancy and early childhood are likely to be diagnosed as being at risk for ADHD when entering elementary schools [30]. Similarly, machine learning and a wearable biosensor help to predict imminent aggressive behavior in inpatient young people with ASD [27]. In addition, several robot-based ADHD screening devices have been tested, such as a contactless sensing system, a deep learning–based classifier, storyboard content for children, and an automated childhood ADHD classifier [29,31,32]. The contactless sensing system, for instance, quantitatively measures the movements of children with ADHD [29]. These devices automatically detect and analyze behavioral reactions, and identify results based on collected data [29,31,32].

Furthermore, machine learning and robot-based devices are also applied to interventions. Remote robot–based interventions are effective in enhancing the level of concentration and encouraging positive learning attitudes among children with severe ADHD symptoms [28]. They recognize a robot as a peer, a good behavioral model, and a learning helper [28].

The overall observations and findings imply that robot-based models are relatively more attractive in younger ages. Machine learning systems also have a higher effectiveness and accuracy of screening.

#### Gaming and Visual Contents

Many types of gaming can be a part of digital therapeutics, depending on their medium. Given that a gaming approach has less rejection than others [35], its use is actively discussed in the recent medical device development for ADHD and ASD. Gaming was mentioned in 4 resources. One dissertation [35], 1 interview [33], 1 journal article [34], and 1 research report [36] found their trends in Korean medical device development for ADHD and ASD.

Gaming is applied to various fields today, not just as an entertainment tool [36]. The research found that gaming helps people relieve negative emotions and improves symptoms [36]. The development of gaming items was motivated by one of the limitations that psychiatrists’ diagnoses rely on subjective individual decisions [33]. A gaming device, AttnKare, made by Hippo T&C, is complex equipment that uses a VR test and measures eye movements and patience [33]. The AI in the device analyzes the collected information and makes individual diagnoses [33].

The cognitive rehabilitation field recently uses computer technology, focusing on basic cognitive function, memory, problem-solving ability, and perception of space and time [35]. This new digital model is personalized to different individuals [35]. Serious games in this field, defined as education-purposed games with entertaining functions [36], are a promising method that can result in easier and faster positive outcomes both in education and treatment [35]. For example, when comparing responses from 2 groups using a communication-functional board game or a traditional board game, those who used a communication-functional board game showed a better score in self-control, self-esteem, family function, and peer relationship [34].

#### Eye-Feedback and Movement Intervention

Eye-feedback and movement technologies are often found in ADHD screening devices. Because eye movements are linked to brain areas with neuropsychological functions, such as response inhibition, selective attention, and working memory, their impairments lead to the primary traits of ADHD [41]. Eye-feedback and movement intervention also have a complex relationship with the categories above, such as VR and AI, gaming, and machine learning. The information about this category was found in 5 resources. Two dissertations [37,38] and 3 journal articles [39-41] described medical devices using eye-feedback and movement intervention.

Using a screening model for ADHD with eye-tracking features and machine learning, 33 eye-tracking features were identified to distinguish children with ADHD from developing children [41]. Eye-tracking characteristics have the potential to serve as a reliable marker for compromised neurobiological function in individuals undergoing ADHD screening [41]. The focus reaction time tests were identified as a valid tool for diagnosing children with ADHD [40]. Given that visual materials tend to be eye-catching and vision accounts for 80% of human recognition [37], visual content can also play an important role in developing interventions for ADHD using eye movements. For example, eye-feedback training improves sluggish cognitive tempo, one of the symptoms of ADHD that shows a lack of energy, slowness in behavior or thinking, and drowsiness [38]. A motion-training system with real-time visual feedback also facilitated motion control in children with ASD [39].

#### Electroencephalography and Neurofeedback

This category discusses electroencephalography and neurofeedback. Both concepts are relevant to digital technologies, including gaming and machine learning [49]. Electroencephalography, a recording of the brain’s electrical activity, measures brainwaves. Neurofeedback is used to modify brainwaves by providing stimulus in neurofeedback training, which is considered a promising physiological approach for the diagnosis and interventions of neurological disorders, such as ASD and ADHD [42,45,47,48,52-56,60]. This topic was mentioned in 19 resources. Two research reports [44,45], 10 journal articles [42,43,48,50,51,55,56,58-60], and 7 dissertations [46,47,49,52-54,57] discussed electroencephalography-based medical devices.

Wearable wireless systems and sensing systems are new potential solutions for diagnosing ASD and ADHD by collecting physiological indicators [42,43,59]. Electroencephalography can detect the abnormalities of the neural system related to ASD and ADHD [42,43,59]. The research found that ADHD can be diagnosed by sounds derived from brainwaves, using (1) ADHD diagnosing algorithms developed by electroencephalography brainwaves with several mathematical methods, eyes-open, and resting-state brainwaves, and (2) a sonification algorithm to convert brainwaves to musical sounds [46].

Convolutional neural network (CNN) is another emerging idea to automatically extract electroencephalography features for medical diagnosis [48,49]. CNN is a neural network modeled after the functioning of the visual cortex for processing data that contains spatial information [49]. Recent research explores deep learning–based devices using CNN to effectively classify electroencephalography signals [48]. A deep learning–based approach using functional magnetic resonance imaging (fMRI) was also another recent discussion [49]. While previous trials covered the entire brain area to identify ADHD, the recent study suggests examining specific brain portions related to the classification of ADHD using the deep learning system by demonstrating a higher level of accuracy [49].

Neurofeedback is another key topic in interventions for ADHD and ASD. Neurofeedback training is a form of self-regulation therapy for brainwaves, using the concept of operant conditioning [52]. During brainwave measurement, patients receive visual or auditory feedback on cortical activity to normalize brain function by inhibiting or reinforcing specific frequency ranges of brainwaves [52]. Neurofeedback enables them to receive real-time feedback on their brainwave states and engage in training to regulate brainwaves as desired [52]. Many studies claim that neurofeedback training positively impacts children with ADHD [47,52-54,60].

Neurofeedback training positively impacted children with ASD by improving their attention and abnormal brainwaves [52]. Students who received neurofeedback training showed increased scores in memorizing numbers and matching colors, numbers, and words [52]. Furthermore, neurofeedback training can be applied to those with ADHD [47,60]. Recent research has reported that 30% of people with ADHD with executive function deficits and inhibitory deficits cannot be treated both by medication and CBT [50,60]. Neurofeedback training is suggested as one of the promising alternative solutions of medication to improve executive functions, inhibition, and working memory [47,50,60]. Moreover, delayed language development and communication ability among children with ADHD can be improved by neurofeedback training [53,54]. In fact, parents having children with ADHD have reported positive outcomes after using neurofeedback training [50]. These trends imply that neurofeedback models can be more effective when they are integrated with different digital items, such as VR, gaming, and AI [54].

As an example of complex medical devices for interventions for ADHD, research suggested a robotic intervention education using neurofeedback [55]. In this program, students with ADHD were encouraged to craft a robot and control its movements using brainwave signals [55]. This program aimed to enhance the level of concentration as well as treatment of ADHD with a children-level storyline [56]. The satisfaction was evaluated positively, while a general operation process had a few comments on further development [56]. In addition, CNN is also used to diagnose ADHD in young children <16 years who are too immature to perform self-diagnosis or use medical equipment [51]. Gaming content is used to increase the objectivity and accuracy of ADHD diagnosis and collected electroencephalography data are classified based on the CNN model [51].

With a similar context of education using neurofeedback, the Korea Institute of Curriculum and Evaluation conducted a 2-year project to design and implement brain-based training for children with ADHD [44,45]. Neuroeducation was applied to the project to explore neuroeducational research tools, including electroencephalography, positron emission tomography, and fMRI [44]. fMRI was especially highlighted to indirectly measure brain activity status by quantifying cerebral blood volume, cerebral blood flow, and blood oxygen saturation [44]. The training program, named Korea Institute of Curriculum and Evaluation Working memory Enhancement Program, involves altering brain function through interaction with the environment, which leads to improved cognitive functions [44,45]. The Korea Institute of Curriculum and Evaluation Working memory Enhancement Program showed positive outcomes among children with ADHD in a clinical trial by enhancing cognitive abilities and demotivating behavioral problems [45].

#### Traditional Therapy

Traditional therapy mostly does not use medical devices. The 3 categories under traditional therapy examined the trends in traditional treatments and diagnosis for ADHD and ASD.

#### CBT and Working Memory

CBT and working memory fundamentally aim to improve cognitive ability as well as attention deficits and impulsive behaviors [61,63,64]. CBT focuses on a behavioral intervention [61], while working memory refers to a cognitive function that involves temporarily holding or manipulating information for a short period [62,63]. They were found in 5 journal articles [61-65].

CBT can be a more effective intervention for adults with ADHD than children with ADHD because adults relatively have a higher cognitive ability and reflective thinking [61]. CBT demotivated people to think about ADHD and think negatively, while knowledge of ADHD was increased [61]. CBT can also be developed as a self-monitoring cognitive training program to help children with ADHD regulate and monitor their thoughts and behaviors during task execution [64]. This approach focuses on individual behavioral problems as well as individual thinking processes, which can be applied to diverse treatments and research on ADHD [65].

A working memory training program is another method to reduce impulsive behaviors and hyperactivities [63]. Given that delivery forms of information and cognitive ability are correlated with one another, previous research findings indicated that delivery forms of information influence outcomes of working memory training programs [62]. This statement implies that a better performance is presented when performing a preferred delivery form of information [62], meaning that understanding a target population’s preference for communication matters in working memory training.

#### Diagnosis and Rating Scales

While many studies discuss the recent trends in medical devices for ADHD and ASD, mostly focusing on digital technologies, traditional methodologies of diagnosis and rating scales are still discussed to update the standards and guidelines. The diagnosis and rating scales were examined in 10 resources. One news release [73], 7 journal articles [1,66,68-72], and 2 dissertations [67,74] addressed the current trends in diagnosis and rating scales of ADHD and ASD.

ADHD diagnosis should be systematically approached through diagnostic algorithms to make safe and accurate decisions [70]. Multiple factors, including age, gender, and individual perceptions of ADHD, need to be considered, and the diagnostic decision needs to be based on the *Diagnostic and Statistical Manual of Mental Disorders, 5th Edition* (*DSM-5*) [70]. The *International Classification of Diseases, 10th Revision* (*ICD-10*) is also discussed [69,70]. Although a few differences are presented between *DSM-5* and *ICD-10*, both models focus on attention deficits, hyperactivities, academic and social difficulties, and impulsive behaviors [70]. ADHD diagnosis usually refers to the *DSM-5*, while public health statistics and materials are based on *ICD-10* [69]. *ICD-10* has more strict standards of ADHD diagnosis than *DSM-5* by recognizing all 3 categories: attention deficits, hyperactivity, and impulsion [69].

In addition to *DSM-5* and *ICD-10*, the Children Behavior Check List (CBCL) is a self-report assessment scale developed to evaluate various emotional and behavioral problems of children and adolescents through reports from parents or close adults in their environment [67]. In South Korea, the US version of the CBCL 4-18 in 1991 was standardized and first introduced as the Korean version of CBCL, and the Korean version of the CBCL 6-18 is the recent version for parents [67,71].

ADHD screening and evaluation were performed in in-person interviews at hospitals, mental-health centers, and school counseling offices [72]. Two interview tools are used: Diagnostic Interview Schedule for Children-IV and Kiddie-Schedule for Affective Disorders and Schizophrenia-Present and Lifetime Version (K-SADS-PL-K) [72]. While Diagnostic Interview Schedule for Children-IV is a structured interview tool that can be administered by general people, K-SADS-PL-K is a semistructured tool that should be administered by trained evaluators [72]. With K-SADS-PL-K, a recent study tried the advanced test of attention, consisting of visual tests and auditory tests that present target and nontarget stimuli at regular intervals, and participants were instructed to respond only to the target stimuli [74]. However, the accuracy of distinguishing a group with ADHD from another group without ADHD was not high, which suggests limitations in using the advanced test of attention as a diagnostic tool for confirmation [74].

Given that ADHD symptoms tend to be presented at an early age, parents’ and teachers’ knowledge and perception of ADHD greatly impact their children’s diagnosis and intervention [1]. Interestingly, the ratings of parents and teachers about symptoms of children with ADHD had no significant correlations, and parents’ ratings and DISC were not matched [1]. By contrast, the rating of teachers was consistent and showed a high correlation with DISC [1]. These findings imply that DISC and the rating of teachers are more reliable and consistent than the rating of parents [1].

In case childhood ADHD may persist into adulthood, the Korean Adult ADHD Rating Scale was developed for monitoring and screening treatment of adults with ADHD [66]. Inattention was recorded as the most general symptom of ADHD in adulthood [66]. The Korean Adult ADHD Rating Scale is expected to effectively rate difficulty in emotional control and disorganization, such as inattention, hyperactivity, and impulsivity [66]. This rating scale was also suggested to extend its range of use to adolescents, embrace gender differences, and identify screening and rating scales, respectively [68]. In this light, traditional rating scales are consistently developed. For example, one of the recent rating scales is a tactile stimulation distribution device to quantify exercise and MBSR [67]. The details of MBSR and other traditional therapies are discussed in the last category.

#### Musical, Literary Therapy, and MBSR

MBSR, musical, and literary therapy described here were developed to increase the effectiveness of screening and intervention for children with ADHD. They were addressed in 5 resources. One journal article [77] and 4 dissertations [75,76,78,79] discussed how they were recently shaped.

The tactile stimulation distribution device was motivated by mindfulness, MBSR, and CBT and scientifically demonstrated a level of concentration of subjects [78]. The quantified data of stimulation were compared with the cognitive outcomes of subjects [78]. The correlative data were referred to as concentration, and the opposed data were considered a distraction [78]. This logic was also supported by left- and right-brain activities [78]. In fact, an MBSR-based program showed a significant improvement in reducing inattention, stress, and anxiety in college students with ADHD [78].

Another approach to intervention for children with ADHD is literary therapy based on social skills training [78]. The program was designed to train them to improve a social relationship between peers and adults and engage in group activities at home and school [78]. Using photo cards and photo books to inspire their imagination and creativity, the general symptoms of ADHD decreased with a significant improvement in emotional and mental stability [78].

Musical therapy is also used for screening and intervention of ADHD. Screening ADHD using musical therapy aims to strategize a plan of treatment by understanding individual conditions and the goals of treatments [79]. This screening is essential to comprehend how musical reactions can be used to improve symptoms when music attracts clients’ changes [79]. While musical therapy screening is designed for a broad understanding of individuals, interventions using musical therapy have a specific purpose to target specific symptoms. A rhythm-based musical intervention was developed to enhance timing control in children with ADHD [75]. The protocol contributed to controlling motor timing and perceived timing using a metronome, guiding a proper speed of response to suggested stimuli in the environment [75]. Carl Orff’s pedagogics, focusing on improvisatory performance with observation, imitation, exploration, and music literacy, is another type of intervention using musical therapy [76]. This program required small group activities, which encouraged social interaction with different individuals [76]. Furthermore, improvisatory work improved inattention, and imitating musical expression demotivated hyperactivities [76]. These findings indicate that musical therapy is a highly effective method both for screening and intervention in children with ADHD.

## Discussion

### Principal Findings

This study conducted a review of the literature to reduce gaps in the research related to medical device use and development in South Korea for the diagnosis and management of ADHD and ASD. The trends in Korean medical device development for ADHD and ASD are categorized into 2 major groups with 8 subgroups in total. Digital therapeutics using AI, machine learning, and electroencephalography technologies account for the biggest portions of development in South Korea, rather than traditional therapies. Given that both ADHD and ASD are neurological disorders, emerging medical device technologies especially focus on electroencephalography and neurofeedback. Different types of digital models are combined or applied to understand brain activities and brainwaves.

In this vein, future development of medical devices for ADHD and ASD is predicted to heavily rely on digital technologies. As digital medical devices are emerging trends in South Korea, they can also be integrated with traditional therapies. For example, the rhythm-based musical intervention can be applied to a gaming device for ADHD, which can also detect particular brainwaves and provide real-time neurofeedback. Recent research has reported that traditional therapies, including musical features and MBSR, have succeeded in screening and intervention for ADHD and ASD. Understanding their strengths and integration with digital medical devices will double the effectiveness of screening and intervention outcomes.

However, this growing transformation is faster than people’s perception of their development. To follow the trends and learn digital literacy for new digital medical devices, training programs about up-to-date digital devices for ADHD and ASD are recommended, especially for parents and teachers to relieve tension in school. The active application of digital devices in school settings is also expected to enable early diagnosis and treatment for students with ADHD or ASD. Because parents and teachers are primary and secondly important people for children with ADHD or ASD [1], education for them is essential to implementing new medical devices into routine care in the real world.

In addition to the application of digital devices, traditional therapies are used for children with ADHD or ASD in school settings. While digital therapeutics is a promising tool today, traditional therapies have still demonstrated their efficacy in screening and interventions. The research presented real-world case studies of the applications that showed positive outcomes and high reliability [75,76,78,79]. Extending this idea, future research could discuss the potential efficacy of integrating digital therapeutics and traditional therapies for the diagnosis and interventions of ADHD and ASD. Furthermore, potential ethical dilemmas associated with the use of medical devices for these conditions are another important topic to study. Understanding the negative effects and limitations of different types of devices in clinical settings will also guide the direction of future development of medical devices for ADHD and ASD.

### Limitations

The first limitation of this study is that many resources had small population sizes to conclude their findings, which makes it hard to generalize the outcomes. To define the accurate trends in Korean medical device development for ADHD and ASD, additional studies conducted with larger populations should be examined. Second, a lower number of records specifically discussed medical devices for ASD, while most of the selected resources focused on ADHD. The results had to focus more on devices for ADHD than ASD. Further research on medical devices for ASD should be studied to understand the need for medical devices for ASD. These studies expect to promote early diagnosis and interventions, which lead to reduced prevalence rates for both ADHD and ASD. Third, given several emerging medical device areas, most of the selected resources were dissertations. They helped understand the recent trends in medical devices for ADHD and ASD; however, peer-reviewed journal articles are required in the future to examine in-depth trends in specific medical devices for ADHD and ASD. Fourth, the limited number of databases were used, especially only 1 Korean database was explored. Fifth, search terms are difficult to truly replicate the same search in the different languages. Further research is recommended to conduct Korean-focused medical devices by directly communicating with Korean medical device companies and relevant experts to reduce the language gaps. The results from this paper will help guide future works.

### Conclusions

In conclusion, this study aims to provide significant insight to understand the recent trends in Korean medical device development, focusing on medical devices for ADHD and ASD. Emerging digital medical devices and those integrated with traditional therapies are some of the important solutions to reducing the prevalence rates of ADHD and ASD in South Korea by promoting early diagnosis and intervention. Furthermore, their application will relieve pressures on teachers and school-based special education programming by providing direct supporting resources to students with ADHD or ASD. Educating parents and teachers about the trends in relevant medical devices also matters in further responses to their children. Further research is recommended to focus on medical devices for ASD given that the number of current studies discuss those for ADHD rather than ASD.

## Appendix

Multimedia Appendix 1 Search strategies and eligibility criteria.

## Abbreviations

ADHD: attention-deficit/hyperactivity disorder
AI: artificial intelligence
AR: augmented reality
ASD: autism spectrum disorder
CBCL: Children Behavior Check List
CBT: cognitive behavioral therapy
CNN: convolutional neural network
DSM-5: Diagnostic and Statistical Manual of Mental Disorders, 5th Edition
fMRI: functional magnetic resonance imaging
ICD-10: International Classification of Diseases, 10th Revision
K-SADS-PL-K: Kiddie-Schedule for Affective Disorders and Schizophrenia-Present and Lifetime Version
MBSR: mindfulness-based stress reduction
VR: virtual reality
