# Supplementary material for: Trends in South Korean Medical Device Development for Attention-Deficit/Hyperactivity Disorder and Autism Spectrum Disorder: Narrative Review
Source: JMIR Biomed Eng. 2024 Oct 15;9:e60399. doi: 10.2196/60399 (PMC11522664; doi:10.2196/60399)
Supplement: Multimedia Appendix 1 [file biomedeng_v9i1e60399_app1.docx]

**Multimedia Appendix 1**

**Table of Contents**

[**Section 1: Search strategies** 2](#_Toc150806577)

[**Section 2: Eligibility criteria for study inclusion** 3](#_Toc150806578)

# **Section 1: Search strategies**

**PubMed Search**

Date of search:8/4/2024

| # | Search | Results |
| --- | --- | --- |
| 1 | ("ADHD" AND “medical device”) OR (“ASD” AND “medical device”) AND (“early” OR “screening” OR “training” OR “treatment” OR “diagnosis” OR “intervention”) | 22 |

**National Assembly Library Search**

Date of search: 8/4/2024

| # | Search | Results |
| --- | --- | --- |
| 1 | (adhd & 의료기기) or (자폐 & 의료기기) & (진단 or 훈련 or 치료 or 검사) | 1,689 |

**Scopus Search**

Date of search: 25/7/2024

| # | Search | Results |
| --- | --- | --- |
| 1 | ("ADHD" AND “medical device”) OR (“autism” AND “medical device”) AND (“early” OR “screening” OR “training” OR “treatment” OR “diagnosis” OR “intervention”) | 56 |

**PsycINFO Search**

Date of search: 26/7/2024

| # | Search | Results |
| --- | --- | --- |
| 1 | ("ADHD" AND “medical device”) OR (“autism” AND “medical device”) AND (“early” OR “screening” OR “training” OR “treatment” OR “diagnosis” OR “intervention”) | 27 |

# **Section 2: Eligibility criteria for study inclusion**

|  | **Inclusion criteria** | **Exclusion criteria** |
| --- | --- | --- |
| Publication date | Published in 2013 or later | Published before 2013 |
| Language | Written in Korean or English | Written other than Korean or English |
| Relevant topics | Korean medical equipment for ADHD and ASD | Not discussed at least one topic about medical device for ADHD and ASD, or not connected to the research objective |
| Subjects | Medicine, neuroscience, computer science, engineering, psychology, social sciences, health professions | Arts and humanities, pharmacology, toxicology, pharmaceutics, mathematics, veterinary, nursing, materials science, economics, econometrics, finance, dentistry, chemical engineering, biochemistry, genetics, molecular biology, immunology, microbiology |
